# FEELING YOUNGER TODAY? THE ROLE OF MEANINGFUL AND FLOW-CONDUCIVE ACTIVITIES

**DOI:** 10.1093/geroni/igad104.1537

**Published:** 2023-12-21

**Authors:** Dwight Tse

**Affiliations:** University of Strathclyde, Glasgow, Scotland, United Kingdom

## Abstract

Given the conceptualization of subjective age being a construct that varies daily, one question naturally follows is whether doing particular activities may be associated with daily subjective age. Specifically, extrapolating from flow and vital engagement theory, we hypothesize that activities that are personally meaningful or flow-conducive (activities that are conducive to flow experience, a psychological state of simultaneously intense concentration and enjoyment) may be associated with older adults’ feeling younger on the day. In a 14-day diary study, participants residing in the United Kingdom reported their subjective age at the end of the day. They also identified the activity on which they spent most time during the day and reported their flow experience and perceived meaningfulness in the activity. With the initial data from a sample of N = 69 adults (age range: 50 to 83) who completed at least 7 daily surveys, we found that higher-than-usual (i.e., within-person) levels of both activity meaningfulness and flow experience were associated with feeling younger. These effects were robust even after controlling for daily stressors and uplifts. Interestingly, there was an interaction between within-person activity meaningfulness and flow experience, such that the effect of flow experience was stronger in activities with lower (vs. higher) levels of meaningfulness. Our findings provide preliminary support that doing personally meaningful and flow-conducive activities may be associated with feeling younger on the day, underscoring the possibility of activity-level behavioral change that influences subjective age.

